# Aquatic Respiration Rate Measurements at Low Oxygen Concentrations

**DOI:** 10.1371/journal.pone.0089369

**Published:** 2014-02-19

**Authors:** Moritz Holtappels, Laura Tiano, Tim Kalvelage, Gaute Lavik, Niels Peter Revsbech, Marcel M. M. Kuypers

**Affiliations:** 1 Department of Biogeochemistry, Max Planck Institute for Marine Microbiology, Bremen, Germany; 2 Department of Bioscience, Microbiology, Aarhus University, Aarhus, Denmark; 3 Department of Oceanography, Dalhousie University, Halifax, Nova Scotia, Canada; French Blood Institute, France

## Abstract

Despite its huge ecological importance, microbial oxygen respiration in pelagic waters is little studied, primarily due to methodological difficulties. Respiration measurements are challenging because of the required high resolution of oxygen concentration measurements. Recent improvements in oxygen sensing techniques bear great potential to overcome these limitations. Here we compare 3 different methods to measure oxygen consumption rates at low oxygen concentrations, utilizing amperometric Clark type sensors (STOX), optical sensors (optodes), and mass spectrometry in combination with ^18-18^O_2_ labeling. Oxygen concentrations and consumption rates agreed well between the different methods when applied in the same experimental setting. Oxygen consumption rates between 30 and 400 nmol L^−1^ h^−1^ were measured with high precision and relative standard errors of less than 3%. Rate detection limits in the range of 1 nmol L^−1^ h^−1^ were suitable for rate determinations in open ocean water and were lowest at the lowest applied O_2_ concentration.

## Introduction

The oxygen concentration in the ocean interior depends on a balance between oxygen consumption and oxygen transport from surface waters, where oxygen is produced and exchanged with the atmosphere. Hypoxic and anoxic zones appear usually where oxygen transport is reduced and/or oxygen consumption is increased. Reduced ventilation is the main cause for anoxia in semi enclosed seas such as the Baltic Sea [1] and the Black Sea [2], whereas a combination of sluggish ventilation of upwelling source waters and high organic matter input causes the development of oceanic oxygen minimum zones (OMZs) at the western continental margins [3] and the Arabian Sea [4]. Severe oxygen depletion has serious consequences for aquatic ecosystems [5] and can cause massive mortality of benthic and pelagic macro-fauna. It is expected that OMZs will expand as anthropogenic pressure increases, and regions with already low oxygen will be most affected [6]. Many predictions are either based on historical data of OMZ volumes that are extrapolated into the future [7] or they are based on ocean circulation models that predict a decline in oxygen transport caused by lower oxygen solubility and increased stratification [8]. Since the transport of oxygen is directly linked to the advection and mixing of water masses well established oceanographic approaches can be applied to predict future changes of oxygen transport. Oxygen consumption – the sink term in the O_2_ balance - depends on biotic processes, such as organic matter availability, the abundance and composition of heterotrophic communities, and oxidation rates of reduced compounds (e.g., NH4^+^ and H_2_S). So far, estimates of oxygen consumption in marine waters are difficult to obtain. Basin scale estimates can be derived from particulate carbon flux modeling [9], or time integrated oxygen consumption is back-calculated from the assumed residence time of water masses and the oxygen deficit, i.e. the apparent oxygen utilization (AOU) [3]. However, validation of these estimates with experimentally measured oxygen consumption rates is scarce. A reason for the lack of experimental data is that incubation experiments require highly precise O_2_ measurements to detect significant consumption rates in the range of a few nmol L^−1^ h^−1^ within reasonable short time intervals up to 24 hours. Long term incubations over several days are strongly biased by so called bottle effects that usually enhance bacterial growth [10]. Time series of Winkler titrations, the classical method to measure O_2_ concentrations, are often applied to study primary production and respiration in the euphotic zone [11]. However, consumption rates in the upper mixed layer are considered to be several fold higher compared to those in the aphotic zone [9] where a high number of incubation replicates may be needed to detect significant rates. Even more challenging is the investigation of oxygen dynamics at the low O_2_ concentrations found in OMZ waters. It requires a low detection limit of less than 1–2 µmol L^−1^, which is the current limit of common methods used in oceanographic surveys such as Winkler titration, electrochemical and optical sensors [12]. A low detection limit is particularly necessary to study (i) the kinetics of the various oxygen consuming processes such as heterotrophic respiration and ammonium and nitrite oxidation; (ii) the inhibitory thresholds of anoxic processes such as anammox and denitrification; (iii) and the interaction of these oxic and anoxic processes.

The recent development of the amperometric STOX sensor [13] lowered the detection limit to a few nmol O_2_ L^−1^ by applying frequent in situ zero calibrations of the sensing cathode. The STOX sensor was successfully applied to measure insitu oxygen concentrations in the OMZ off Chile and Peru [14]–[16], off Mexico (L. Tiano et al. unpubl. results), and in the Arabian Sea [17]. In parallel, the optical oxygen sensors (optodes) were recently improved with respect to precision and sensitivity by the development of special dyes for trace amounts of oxygen ranging from 0–50 µmol L^−1^. In general, the precision of optodes increases with decreasing oxygen O_2_ concentrations [18], which makes them well suited for rate measurements at low concentrations. A third approach for highly sensitive and precise O_2_ measurements is the detection of the stable oxygen isotope ^18-18^O_2_ by membrane inlet mass spectrometry (MIMS). The low natural abundance of ^18-18^O_2_ (0.2%) and the very low cross-sensitivity of mass 36 (^18-18^O_2_) with other dissolved gases allows to follow its consumption over time upon the initial addition to the incubated sample. To evaluate the applicability of all three approaches (STOX, optodes and ^18-18^O_2_ labeling), we performed combined incubation experiments measuring O_2_ consumption in water samples with low oxygen concentrations of 0.5–15 µmol L^−1^.

## Methods

### STOX Sensor

The electrochemical STOX sensor measures oxygen partial pressure. Different from conventional oxygen microsensors, the STOX sensor features a second cathode - a porous front guard cathode - that prevents O_2_ to diffuse to the sensing cathode when polarized. Switching on and off the front guard cathode allows for in situ zero calibration of the sensor and thereby lowers the detection limit to less than 10 nmol L^−1^ [13]. Electrochemical sensors consume oxygen at a rate proportional to the measured O_2_ concentration. At concentrations used in this study (1–15 µmol L^−1^) the O_2_ consumption rate of the STOX sensor is between 0.012 to 0.18 nmol L^−1^ h^−1^ and can therefore be neglected. The microsensors for this study were built as described previously [13], [19]. The sensor currents were measured with a PA8000 eight-channel picoammeter (Unisense A/S, Denmark), while the polarization and depolarization of the front guard were regulated by a custom-built timer-controlled switchbox with the timer set to 190–200 s intervals for front guard on and off, respectively. The signals were processed and recorded by a Unisense ADC816 16-bit A/D converter, connected to a portable PC using the program Sensortrace Basic (Unisense A/S).

**Figure 1:**
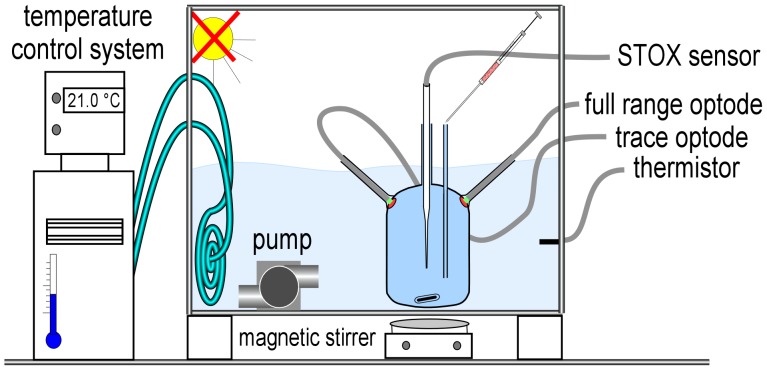
Incubation setup for Experiment 1. The incubation bottle was placed in a temperature controlled water bath. The 2 optodes (full range and trace) were placed on opposite sides in the upper half of the bottle. The STOX sensor was inserted through a glass tube in the center of the bottle. For calibration, known volumes of air saturated water were added through the pressure compensation port. A glass coated magnet was rotated by a magnetic stirrer below the container.

Incubation experiments using the STOX sensors have been carried out in modified Schott Duran glass bottles of 1160 ml volume (Fig. 1). The incubation bottle had two openings, one to insert the STOX sensor and another one to allow pressure compensation for temperature induced volume changes. Oxygen transport through both openings was limited to negligible amounts by diffusion through a narrow passage over a long distance. The pressure compensation port consisted of a 250 mm long glass tube penetrating the glass wall of the incubation bottle. Because of the small inner diameter (3 mm) the water inside remains stagnant and oxygen transport is possible only by means of molecular diffusion. However, the inner diameter was wide enough to allow the injection of additives using a hypodermic needle. The STOX sensor was inserted through the second port consisting of a 30–40 mm long glass tube. The distance between senor casing and the inner wall of the glass tube (I.D. 8 mm) was within 0.1 mm. During the incubations the bottles were kept in the dark and submersed in a temperature controlled water bath at ∼21°C. Continuous stirring was applied using 2.5-cm long glass coated magnets (Fisher Scientific) at 30–60 rpm driven by magnetic stirrers (IKA) placed underneath the water bath. The stirring sensitivity of the STOX sensors was approximately 7% [13]. The stirring sensitivity is defined as the change of O_2_ reading in stagnant water versus vigorously stirred water. Considering that there is already a constant stirring in the incubation bottle a change of stirring speed would result in a concentration change much smaller than 7%.

### Optode Spots

Optical oxygen sensors (optodes) are made of oxygen sensitive fluorescent dyes where fluorescence intensity and lifetime depends on oxygen partial pressure [18]. Optodes are widely used in aquatic research, often to monitor O_2_ concentration over longer time periods, taking advantage of their mechanical stability and long lifetime [20], [21]. In contrast to the electrochemical STOX sensor, optodes do not consume oxygen and are therefore not stirring sensitive. In this study we used non-invasive oxygen sensor spots for O_2_ concentrations ranging between 0–1400 µmol L^−1^ (full range spot, PST6) and 0–57 µmol L^−1^ (trace spot, PST3) together with the corresponding fiber optics and electronics from PreSens (Precision Sensing GmbH, Germany). The optode spots (diameter 4.5 mm, thickness 0.2 mm) were fixed to the inner surface of the STOX incubation bottle (Fig. 1) using acetic acid curing silicone rubber. The silicone was allowed to cure for 2 days at room temperature before the experiment. The fluorescent signal of the sensor spots was read from outside the bottle with optical fibers. The fibers were positioned within a plastic tube which was fixed perpendicularly to the outside surface of the bottle. The incubation bottle was placed in a water bath that was temperature controlled (21.1°C+/−0.1) to minimize the influence of temperature changes on the optode readings. The water bath itself was placed inside a black plastic container to exclude light penetration. Magnetic stirring was applied as described above for the STOX sensor measurement. Oxygen concentrations were measured with both spots (full range and trace) every 5 s and recorded using the software OxyView 7.01, (© PreSens GmbH).

### ^18-18^O_2_-incubations

With this approach, O_2_ consumption is measured as decrease of initially injected ^18-18^O_2_ over time. Due to the low natural abundance of the stable oxygen isotope ^18-18^O_2_ (∼0.2%) compared to the most abundant oxygen isotope ^16-16^O_2_ (∼99.8%), trace amounts of double-labelled ^18-18^O_2_ (mass 36) can be measured with high accuracy using mass spectrometry.

A saturated (∼1.2 mmol L^−1^) ^18-18^O_2_ stock solution was prepared at room temperatures (20°C) by filling a 12-mL Exetainer (Labco, UK) with He-purged, sterile seawater and replacing 1 mL of seawater with 2 mL of 99% enriched ^18-18^O_2_ gas (Sigma-Aldrich, Germany). The Exetainer was shaken vigorously and stored for ∼24h to ensure equilibration with respect to ^18-18^O_2_.

^18-18^O_2_ -incubations were performed in two different ways. The more general approach was the incubation of a series of 12 mL subsamples (in Exetainers) with the same initial ^18-18^O_2_ concentrations. In this so called Exetainer approach, biological activity was terminated in the subsamples at different time points and the ^18-18^O_2_ concentration was measured afterwards for the entire time series at once, using membrane inlet mass spectrometry (MIMS). Another approach was used to directly compare ^18-18^O_2_ -incubations with STOX sensor measurements. In this so called direct approach, the decrease of ^18-18^O_2_ and total O_2_ over time was determined simultaneously in a single incubation bottle, using parallel STOX and MIMS measurements (see below).

#### The exetainer approach

Water samples were transferred into 250-mL serum bottles and purged with helium (He) for approximately 15 min to remove all O_2_. The headspace which develops during He-purging was removed by injecting water from a second serum bottle which was treated the same way. With a gas tight syringe, a defined volume of the ^18-18^O_2_ stock solution was added to the serum bottles. To ensure complete homogenization of the ^18-18^O_2_ a glass-coated magnet had been added to each bottle prior to sampling and the bottles were placed on a magnetic stirrer for ∼10 min at ∼450 rpm. From each serum bottle, 12 subsamples were transferred into 12 mL Exetainers using He-overpressure to avoid air contamination (see [22] for detailed description). The Exetainers were incubated in the dark at room temperature (∼23°C). After 0, 1, 2, 4, 6 and 18 hours, biological activity was terminated in two replicate Exetainers by adding 100 µL of saturated mercuric chloride. The concentration of ^18-18^O_2_ in the Exetainers was determined by membrane inlet mass spectrometry (MIMS; GAM200, IPI). The Exetainers were placed upside down and a short needle connected to the membrane inlet of the MS via a gas tight Tygon tubing was inserted into the Exetainers. Using a peristaltic pump, a sample volume of ∼2.5 mL was withdrawn and replaced with He through a longer needle inserted into the Exetainers. The sample was pumped across a gas-permeable membrane with a constant vacuum on its outer side (membrane inlet of the MS). Oxygen consumption rates were calculated from simple linear regression of ^18-18^O_2_ decrease over time, and were corrected for the concentration of residual ^16-16^O_2_ measured in the T0 sample by the MIMS.

#### The direct approach

**Figure 2:**
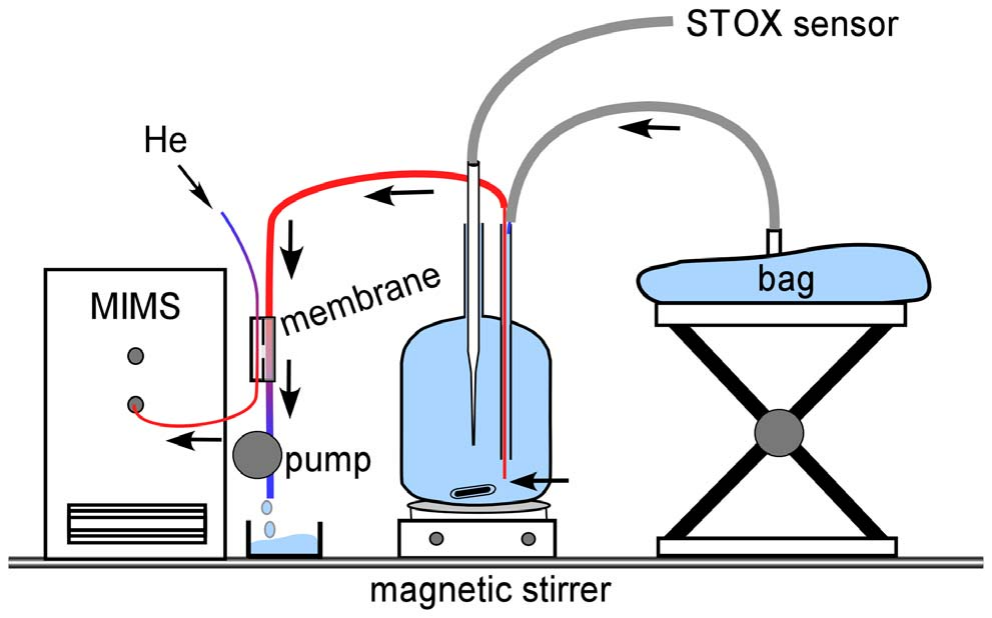
Incubation setup for Experiment 2. Water in the incubation bottle was initially labeled with ^18-18^O_2_. The labeled water was pumped with a flow rate of 0.73 mL min^−1^ out of the bottle and across the membrane inlet of the mass-spectrometer. The water was replaced by degassed unlabeled water from a gas tight bag that was positioned at the upper edge of the pressure port to keep a slight overpressure. Helium was used as carrier gas for stable and fast mass detection.

A STOX incubation bottle (Fig. 2) and a gas tight bag (∼450 mL) made of plastic laminated aluminium foil were filled with the same degassed water. ^18-18^O_2_ stock solution was added to the incubation bottle (kept dark) to a final concentration of 1 µmol L^−1^. A needle was inserted through the pressure compensation port into the bottle to draw subsamples for MIMS measurements (Fig. 2). The removed sampling volume was replaced by degassed water out of the gastight bag which was transferred via a gastight Tygon tubing to the top of the pressure compensation port. Using a peristaltic pump, subsamples for the MIMS were pumped out of the incubation bottle and across a custom-built membrane inlet system with a flow rate of 0.7 mL/min. On the vacuum side of the membrane a constant He flow ensured a stable and fast responding mass detection of the extracted gases. During the incubation, subsamples for the MIMS were pumped for 10 min every 30 minutes and continuously for the last 25 min of the incubation. Of the 10 minute intervals, only the last minute of ^18-18^O_2_ measurements was used for further processing. Concentrations from STOX and MIMS measurement were corrected for the dilution with non-labelled water from the bag.

### Water Sampling and Treatment

#### Ethics statement

No special permission was required to access and sample the water at the following location, and no endangered or protected species were involved during the sampling.

#### Sampling and treatment

Sea water was collected on Dec. 4th 2011 at Randers fjord (56°31′12.22′′N; 10°13′48.59′′E), which is influenced by freshwater discharge (13 ‰ salinity) of two rivers in its inner part [23]. Water was filled into canisters and transferred to the laboratory where it was stored and subsequently used for rate measurements within the following 4 days. In order to obtain very low O_2_ concentrations in the incubation bottles, we applied the following procedure: the water was bubbled for >1 hour with N_2_+0.05% CO_2_ mixture, while enclosed in a 20 L glass bottle. From this reservoir, the incubation bottles were filled using a glass tube siphon with Tygon tubing joints. The filling was performed through the pressure compensation opening of the incubation bottle (Figs. 1 and 2) while a N_2_ gas stream was maintained within the incubation bottle by a 5-mm Tygon tube inserted through the 8-mm sensor opening. The seawater was allowed to overflow for three volume changes before the STOX sensor was inserted. All the glassware was prepared by first washing it with 0.1 M NaOH, and subsequently with 0.1 M HCl to avoid organic contamination.

### Calibration

**Figure 3:**
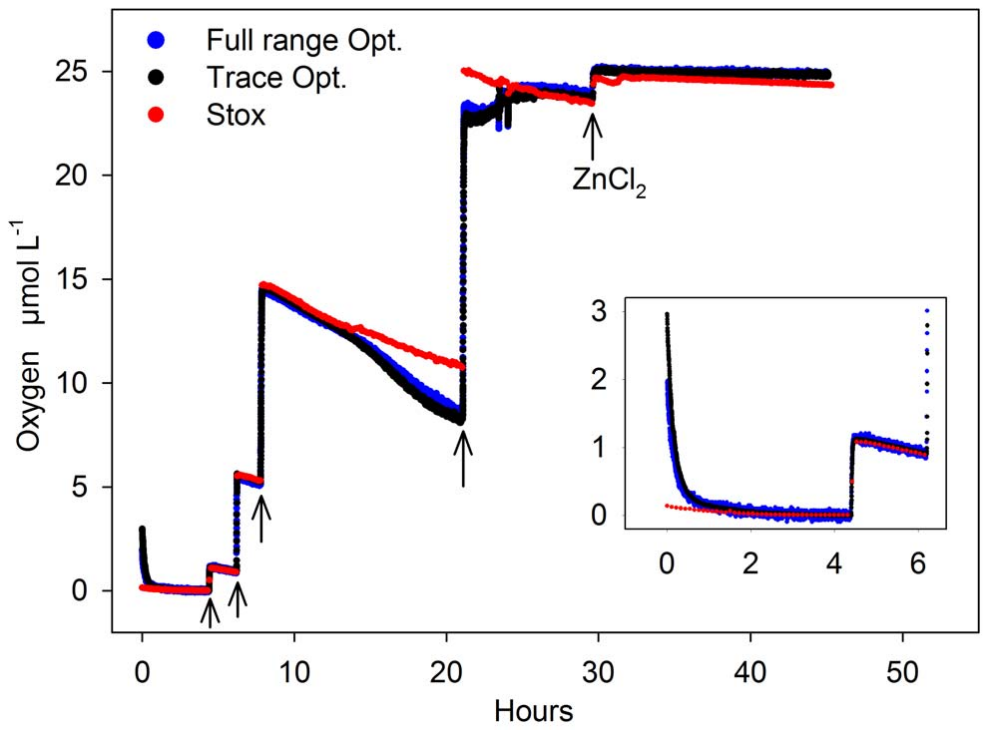
Experiment 1: Comparing optodes (trace and full range) and STOX sensor. Degassed water was successively enriched with oxygen by adding known volumes of air saturated water (arrows). Consumption rates were calculated after each addition from the O_2_ decrease over time (see Figure 4 A–C). ZnCl_2_ was added after 30 hours to stop biological activity. The insert shows the initial adaptation of the optodes in the first few hours.

After filling the incubation bottle with degassed water (see above), the water was incubated for approximately 4 hours until any remaining trace amounts of O_2_ were consumed. We used the in situ zero calibration of the STOX sensor [13] to define zero O_2_ concentration. Zero O_2_ concentration was reached when the differential signal of the STOX sensor was zero. The uncertainty of the zero O_2_ concentration measurement is equivalent to the detection limit of the STOX sensor, which is below 10 nmol L^−1^ [13]. In parallel, zero concentration was indicated by the optodes when the phase shift of the optode sensors showed no further increase (phase shift and O_2_ concentrations are inversely related). The second calibration point was set by injecting known volumes of saturated water into the incubation bottle via the pressure compensation port. The added O_2_ concentration was calculated from the volume of the incubation bottles (1160 mL), the injection volume, and the known O_2_ saturation concentrations at the actual temperature and 13 ‰ salinity according to Garcia and Gordon [24]. A first addition increased the concentration in the incubation bottle from zero to 1 µmol L^−1^ and was used as a second calibration point. Subsequent additions (see Figure 3 below) were used to estimate the uncertainty of this procedure by comparing the O_2_ concentrations measured with the calibrated STOX sensor after the addition with those calculated from the added volumes and the saturation concentration. The error was less than 4.2%. For the MIMS, a two-point calibration was performed based on the ^16-16^O_2_ reading (mass 32) for air-saturated water pumped across the membrane and the ^18-18^O_2_ background signal (mass 36) of the stabilized signal after pump was turned off for >10min.

### Experiments

Three different experiments were set up comparing the 3 different approaches to measure O_2_ consumption rates. In experiment 1, the STOX sensor and 2 optode spots (full range and trace O_2_ concentrations) were compared by measuring O_2_ consumption rates simultaneously in the same incubation bottle (Fig. 1). In experiment 2, O_2_ consumption rates were measured in ^18-18^O_2_ labeled water using simultaneous STOX sensor and MIMS measurements (Fig. 2).

In experiment 3, O_2_ consumption rates were determined from time series of ^18-18^O_2_ labeled subsamples (Exetainer approach) using MIMS. 5 different time series were measured with different initial ^18-18^O_2_ concentrations of 0.6, 0.9, 2.3, 4.4 and 9.3 µmol L^−1^.

### Post-processing and Statistics

For the following rate calculations we assumed zero order kinetics, first of all because the change of concentration used for the rate calculation was usually small, and because half saturation constants for microbial respiration were expected to be in the order of 100 nmol L^−1^ [25] which is below the O_2_ concentrations used for rate calculations in this study. Moreover, any deviation from zero order kinetics is readily detected in the residuals of the linear regression. O_2_ consumption rates were calculated from linear regression of oxygen concentrations over time. A crucial parameter for rate measurements is the precision of the concentration measurement, which is defined as the scatter of repeated measurements around a mean value. Assuming that the true decrease of O_2_ was linear, we used the root mean square of the residuals (RMS_RES_) of the linear regression as a proxy for the precision of the O_2_ measurements. RMS_RES_ was calculated from the difference between observed (C) and predicted (Ĉ) concentrations and the number of individual concentration measurements (n):
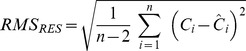
(1)


The standard error of the rate (i.e. standard error of the slope of the linear regression) was calculated from RMS_RES_ and the difference between individual sample time points (t) and their mean (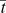):
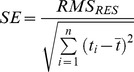
(2)


For a sample size of n>100, the term in the denominator of equation 2 can be approximated from the measuring frequency (F) and the incubation time (T) yielding the simplified equation (see Appendix S1):
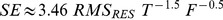
(3)


It is evident from equation 3 that the standard error of the rate decreases linearly with decreasing RMS_RES_, but it also decreases non linearly with increasing incubation time and measuring frequency. The incubation time was different in each experiment, which does not allow a comparison of the standard error between experiments. Moreover, the incubation time was often much less than 24 hours – the maximum incubation length during which bottle effects are assumed less significant. For each incubation we therefore estimated the standard error for an extended 24-hour incubation by inserting the respective measuring frequency and an incubation time (24 h) into Eq. 3. We further assumed that the precision does not change during the incubation, i.e. RMS_RES_ is constant and thus the values from shorter incubations can be applied. Finally, a potential rate detection limit can be defined as two times the normalized standard error. This potential rate detection limit considers only the combined limitation from precision and measuring frequency and constitutes the lowermost detectable rate in case that other limitation such as senor drift can be diminished or excluded.

## Results

### Experiment 1: Comparison of STOX Sensor and Optode Spots

**Figure 4:**
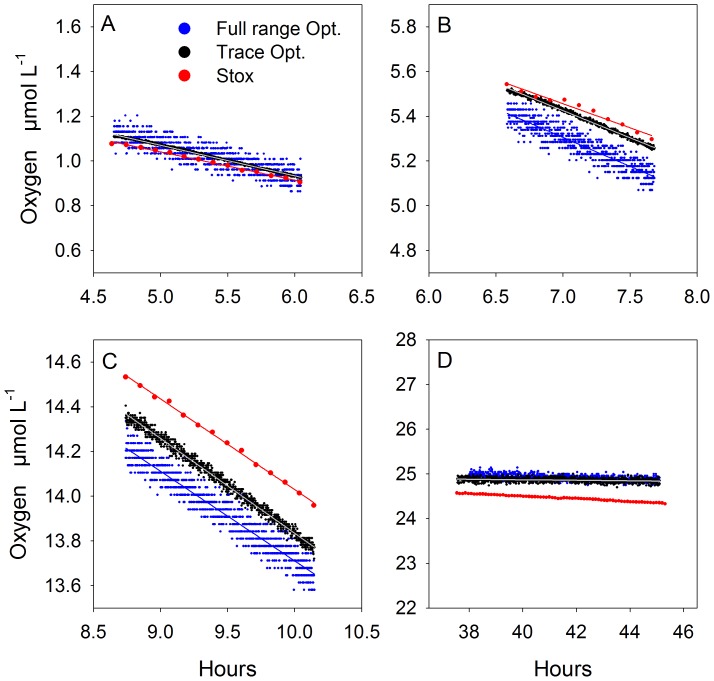
Experiment 1: Comparison of O_2_ decrease over time. The O_2_ consumption measured by optodes and STOX sensor after the adjustment to O_2_ concentrations of 1.2 µmol L^−1^ (A), 5.5 µmol L^−1^ (B) and 14.3 µmol L^−1^ (C), and after the addition of 20 ml ZnCl_2_ (D). Linear fits are indicated by the straight lines. Please note, the aspect ratio of time and concentration axis (µmol L^−1^ to hour) is the same for all figures to allow comparison of slopes.

Experiment 1 lasted for 45 hours (Fig. 3). After the initial addition of degassed sea water, the remaining O_2_ (∼130 nmol L^−1^) was consumed within 4 hours (Fig. 3, insert). Increasing amounts of oxygen were added after 4.5, 6.2, 7.8 and 21 hours (Fig. 3, arrows), each followed by periods of almost constant decrease of O_2_ over time due to O_2_ consumption. Biological activity was terminated after 30 hours by adding 20 ml of 60% (w/v) ZnCl_2_ solution. In general, the oxygen readings of the STOX sensor and both optode spots (full range and trace) agreed well during most of the experiment. Mismatches were found in the first 2 hours, where the optode spots showed increased concentrations due to their initial adaptation to salinity and low O_2_ concentration (Fig. 3, insert). Thereafter, the response to oxygen additions expressed as T_90_ (i.e. the time to reach 90% of the final value) was less than 2.5 minutes. Until hour 14, the offset between STOX sensor and optodes was less than 3% of the O_2_ concentration (e.g. the offset was 30 nmol L^−1^ at a concentration of 1 µmol L^−1^, see Fig. 4). During the night (hour 14–20) both optode spots showed a stronger O_2_ decrease compared to the STOX sensor (Fig. 3). The resulting offset between STOX and optode readings of 2 µmol L^−1^ was maintained during the O_2_ addition at hour 21 but decreased again in the morning and after the stirring speed was increased to ∼100 rpm at hour 23.5. Possible reasons such as microbial growth and biofilm formation are discussed below. In the following the O_2_ time series before the deviation of optode and STOX readings (0–14 hours) and after the addition of ZnCl_2_ are analyzed. Figures 4 A–C show the decrease of O_2_ subsequent to the first 3 O_2_ additions to concentrations of 1.2, 5.5 and 14.3 µmol L^−1^. Consumption rates were calculated from linear regressions for ∼1.2 hour time intervals and increased from 138 to 412 nmol L^−1^ h^−1^ with increasing O_2_ concentrations (Table 1). The offset between the different sensor readings was less than 3%, and the consumption rates calculated from all three sensors agreed well showing a relative standard deviation (i.e. standard deviation normalized by mean value) of less than 10%.

**Table 1:** Summary of measured O_2_ consumption rates, method settings and statistics.

Exp.	Sensor	O_2_ Conc.	O_2_ Rate	SE	RMS_RES_	F	Pot. Det. Lim.
		µmol L^−1^	nmol L^−1^ h^−1^	nmol L^−1^ h^−1^	nmol L^−1^	d^−1^	nmol L^−1^ h^−1^
**1**	Trace Opt.	1.0	138.0	0.24	3.0	17280	0.007
		5.1	242.9	0.70	6.2	17280	0.014
		14	426.0	1.39	17.9	17280	0.039
		24.7 (ZnCl_2_)	5.5	0.69	45.6	2880	0.245
	Full range Opt.	1.0	150.0	2.55	32.2	17280	0.071
		5.1	259.0	4.03	36.0	17280	0.079
		14	402.0	3.59	46.5	17280	0.102
		24.7 (ZnCl_2_)	14.9	0.92	55.2	2880	0.297
	STOX	1.0	126.0	2.57	4.0	222	0.078
		5.1	215.5	12.55	13.5	222	0.261
		14	408.0	5.48	8.6	222	0.166
		24.7 (ZnCl_2_)	29.5	0.34	6.6	222	0.127
**2**	MIMS (direct)	1.0	153.7	3.45	52.4	1860	0.350
	STOX	1.0	158.5	1.96	13.3	191	0.278
**3**	MIMS (Exet.)	2.6	125.6	0.58	29.5	148	0.698
		2.9	126.0	0.93	61.6	148	1.459
		4.2	156.1	1.64	108.7	148	2.577
		6.4	193.2	1.56	79.3	148	1.879
		11.1	203.8	3.06	199.3	148	4.723

The O_2_ consumption rates (O_2_ Rate) measured at different O_2_ concentrations (O_2_ Conc.) and with different sensors and sampling frequencies (F) are summarized. For each rate measurement, standard errors (SE) and root mean square of the residuals (RMS_RES_) were calculated from equations 1 and 2. The potential rate detection limit of an assumed 24 hour incubation (Pot.Det.Lim.) was calculated from equation 3. Please note, a low RMS_RES_ denotes a high precision, and potential rate detection limits are only based on precision and measuring frequency, but do not consider possible limitation from sensor drift or O_2_ contamination.

While the rates and concentrations were nearly identical, the precision of the O_2_ measurement was significantly different between the sensors. The full range optode showed a large scatter of individual measurements with discrete steps of 20–30 nmol L^−1^ (Fig. 4) indicating a limited resolution of phase and temperature readings – the underlying quantities from which O_2_ concentrations are calculated. Consequently, a high RMS_RES_ (Table 1) indicated a low precision of the full range optode, whereas the STOX sensor showed a high precision expressed by low RMS_RES_ (Table 1). Nevertheless, short sampling intervals for the optode measurements (5 s) compared to the STOX measurements (390 s) lead to comparable standard errors of 2.6–12.6 nmol L^−1^ h^−1^ (STOX) and 2.6–4.0 nmol L^−1^ h^−1^ (full range optode), whereas the standard error of the trace optode was up to one order of magnitude lower (0.2–1.4 nmol L^−1^ h^−1^). RMS_RES_ increased with increasing O_2_ concentrations from 32 to 46 nmol L^−1^ and from 3 to 18 nmol L^−1^ for the full range and trace optode respectively, whereas no clear trend was observed for the STOX sensor (4–14 nmol L^−1^). Figure 4 D shows the O_2_ concentration over time after the addition of ZnCl_2_. The consumption rates were strongly decreased but still significant with 5±0.7, 15±0.9, and 30±0.3 nmol L^−1^ h^−1^ calculated from trace optode, full range optode and STOX measurements, respectively.

### Experiment 2: Comparison of STOX and ^18-18^O_2_ Incubations

**Figure 5:**
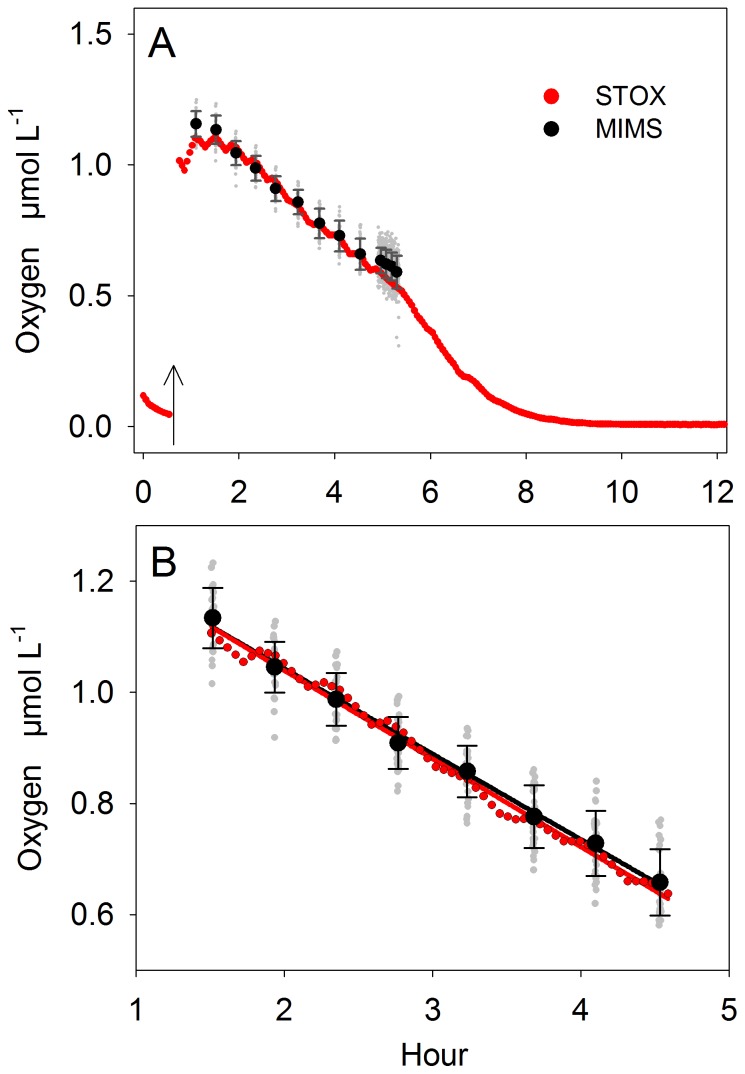
Experiment 2: Comparing MIMS and STOX sensor. O_2_ concentration and consumption rates measured after the addition of 1.2 µmol ^18-18^O_2_ L^−1^ (arrow) (A). Individual MIMS measurements (grey dots) are averaged (black dots) and presented with the respective standard deviation. From the entire experimental run 3 hours of simultaneous measurements (B) were used for rate calculations.

Experiment 2 lasted for 12 hours (Figure 5 A). After the initial addition of degassed sea water into the incubation bottle, O_2_ consumption lowered the remaining trace concentration from 0.11 to 0.045 µmol L^−1^ before ^18-18^O_2_ was added to a final concentration of 1.2 µmol L^−1^ at hour 0.6 (Fig. 5A, arrow). After mixing of ^18-18^O_2_ for 0.4 hours the MIMS measurements started by pumping subsamples for 10 min every 30 min until hour 4.5. O_2_ concentrations were calculated by averaging 30 MIMS measurements taken during the last minute of the pumping interval. After hour 4.5, subsamples where pumped continuously for 24 min and O_2_ concentrations were calculated by averaging 180 MIMS measurements over 6 min. O_2_ consumption rates were calculated from linear regressions over a 3 hour time interval (Figure 5 B).

O_2_ concentration measured by STOX sensor and MIMS were comparable with negligible offset (<3%) resulting in almost identical O_2_ consumption rates of 159±2.0 and 154±3.5 nmol L^−1^ h^−1^, respectively (Table 1). Moreover, the consumption rates were comparable to those measured in Experiment 1 at similar concentrations (1 µmol L^−1^). The direct comparison of the STOX and the MIMS measurement revealed a lower precision of the MIMS measurement which was not fully compensated by its higher measuring frequency and therefore resulted in a higher standard error of 3.45 nmol L^−1^ h^−1^ (Table 1). The dilution of the incubated water during the pumping intervals was an additional source of error. Although the dilution was considered in the calculation, a slight undulation in the decrease of O_2_ was observed by the STOX (Fig. 5B) which increased the standard error of the STOX measurement compared to the one in Experiment 1.

### Experiment 3∶^18-18^O_2_ Incubations in Exetainers

**Figure 6:**
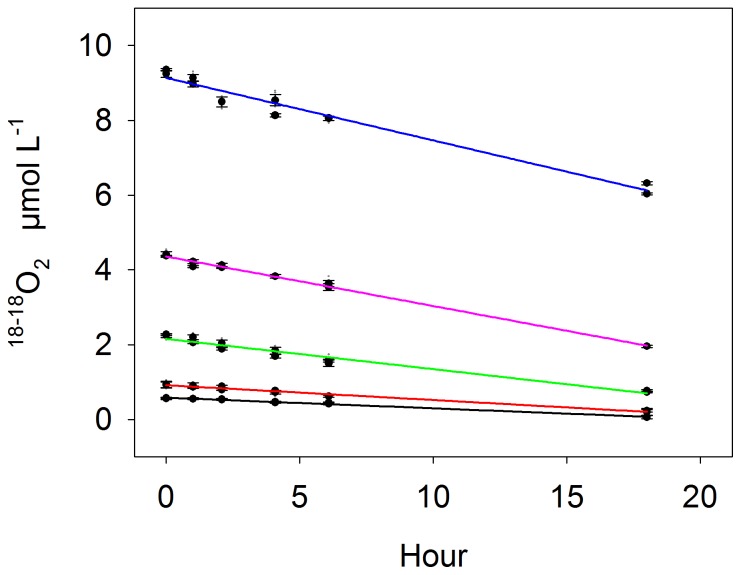
Experiment 3: Time series of ^18-18^O_2_ concentrations in Exetainers. The O_2_ consumption in 5 incubations with different initial ^18-18^O_2_ concentrations was measured. For each Exetainer, individual MIMS measurements are averaged (black dots) and presented with the respective standard deviation.

Five different incubations with initial concentrations of 0.6, 0.9, 2.2, 4.4, and 9.1 µmol ^18-18^O_2_ L^−1^ were run over 18 hours. For each of the incubations, biological activity in 2 Exetainers each was terminated at 6 time points, with an increased resolution of 5 time points over the first 6 hours. In all incubations ^18-18^O_2_ concentrations decreased linearly with time (Figure 6). Residual concentration of ^16-16^O_2_ of 2 µmol L^−1^ were measured in the T0 samples by the MIMS. Assuming negligible isotope fractionation, O_2_ consumption rates were calculated from linear regression of ^18-18^O_2_ concentrations over time and multiplied by the ratio of total O_2_ over ^18-18^O_2_ concentrations measured at the start of the incubation. O_2_ consumption rates ranged between 125±0.6 and 204±3.1 nmol L^−1^ h^−1^ and were increased at high initial O_2_ concentrations (Table 1). The precision of the ^18-18^O_2_ measurement was comparable to those in experiment 2 at similar O_2_ concentrations. However, with increasing O_2_ concentrations the precision decreased and the standard error increased (Table 1).

## Discussion

All three methods - the STOX sensor, both optode spots and the ^18-18^O_2_ incubations - were suitable to detect significant O_2_ consumption rates at low O_2_ concentrations with high precision and a relative standard error of less than 3%. The direct comparison of the methods (Experiments 1 and 2) showed an overall good agreement of O_2_ concentrations and consumption rates. Deviations of O_2_ readings between optodes and STOX were observed in Experiment 1 only between hour 14 and 24 (Fig. 3). The deviation occurred during the night and disappeared in the morning after the addition of oxic water and after the stirring speed was increased. Because the linear decrease of the STOX signal seemed unaffected we assume that both optodes were simultaneously producing the signal offset. The observed signal offset corresponded to a decrease in room temperature of several degrees during the night, which was, however, strongly damped by the water bath to a decrease of less than 0.1°C in the incubation bottles – too little to explain the signal offset. Another possibility is the O_2_ consumption of a slowly growing microbial biofilm on the surface of the optode spots which may lead to a decrease in O_2_ concentrations detected by the spots. The increase of stirring speed at hour 23 could have caused a gradual erosion of the biofilm and thus a response back to unbiased O_2_ concentrations. Although this explanation is speculative and a deviation between optode and STOX measurement was not observed in a previous experiment (data not shown), it emphasizes the potential limitation which may arise from microbial activity and growth, especially in water samples from a shallow coastal environment where microbial abundance and activity is high.

### O_2_ Consumption Rates as a Function of O_2_ Concentrations

**Figure 7:**
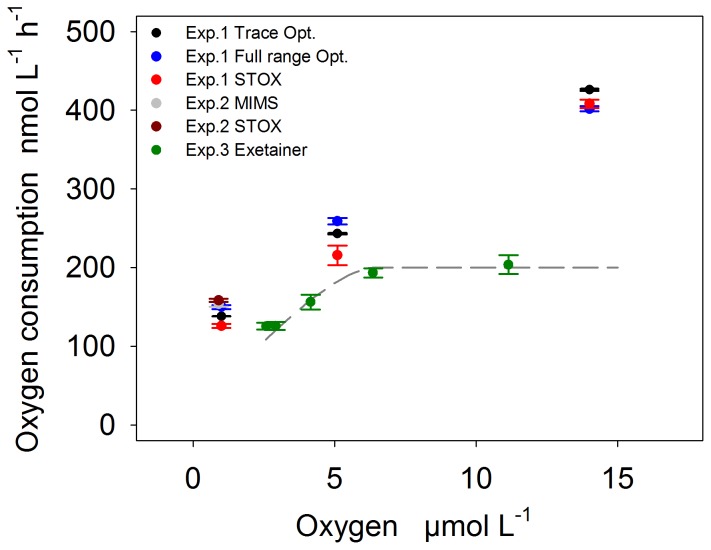
O_2_ consumption rates versus O_2_ concentrations. The O_2_ consumption rates of Experiment 1, 2 and 3 are summarized and plotted over the initial O_2_ concentrations of the respective incubation. Experiment 3: The dashed line represents diffusion limited rates estimated from equation 4 assuming aggregate diameters (r_0_) of 0.14 mm. Please note: rates in Experiment 1 also increase with incubation time and could as well reflect bacterial growth over time.

In Experiments 1 and 3, we observed a significant increase of O_2_ consumption rates with increasing O_2_ concentrations (Figure 7). Between 0–7 µmol L^−1^, a similar trend of ∼25 nmol L^−1^ h^−1^ per 1 µmol L^−1^ was observed for both experiments. In Experiment 1, the O_2_ consumption rates were measured one after the other in the same incubation bottle and at increasing O_2_ concentrations (Figure 3) which also allows interpreting increasing rates as the result of bacterial growth or biofilm formation over time (see above). In fact, considering the O_2_ measurement after the deviation of STOX and optode reading (after hour 25, Figure 3), rates at O_2_ concentrations of 24 µmol L^−1^ were again similar to those at 1 µmol L^−1^ (STOX: ∼150 µmol L^−1^ h^−1^) indicating that the data in Experiment 1 may not illustrate a reliable effect of O_2_ concentrations on respiration rates. In contrast to Experiment 1, the Exetainers in Experiment 3 were incubated simultaneously at different O_2_ concentrations and therefore bacterial growth cannot explain the different rates. Instead, rates could be limited by low O_2_ concentrations. Although half saturation constants for microbial rates are assumed to be in the order of 100 nmol L^−1^ [25], O_2_ consumption rates can be limited already at higher concentrations if the particulate organic matter is aggregated. Inside organic aggregates, the combination of diffusion limited O_2_ transport and high bacterial abundance can lead to the formation of anoxic zones that will increase in volume and decrease the bulk O_2_ consumption rate as the O_2_ concentration outside the aggregate decreases. The diffusion limitation of O_2_ consumption rates depends on the O_2_ demand inside the aggregate, the aggregate size and the thickness of the diffusive boundary layer (DBL) around the aggregate [26]. Below a threshold O_2_ concentration, the O_2_ consumption rate of the entire aggregate decreases with the increase of anoxic volume. By applying and rearranging the analytical solution for solute transport and reaction in a sphere as presented by Ploug et al. [26], the O_2_ consumption rate of an aggregate below a threshold O_2_ concentration can be calculated:
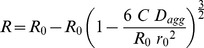
(4)where r_0_ denotes the radius of the aggregate, D_agg_ is the diffusivity within the aggregate, C is the ambient O_2_ concentration and R_0_ is the non limited O_2_ consumption rate of the aggregate. For simplicity we neglected the DBL around the sphere and we assumed zero order O_2_ consumption. In our study, aggregates were observed in the incubated water, but the number and size of aggregates and their specific respiration rates were not analyzed. Instead we use data from a recent study on aggregate distribution in the upwelling region off Mauritania [27] in which O_2_ respiration was related to aggregate diameter by 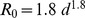 using units of mm (d) and nmol h^−1^ (R_0_). Applying this relation and assuming a diffusion coefficient of 1.3×10^−9^ m^2^ s^−1^, concentration dependent rates of aggregates were calculated from Eq. 4. The shape of this function compares well with the measured decrease of O_2_ consumption rates in Experiment 3, when an aggregate diameter of 0.14 mm is applied in the model (dashed line in Fig. 7). Insitu measurements of aggregate sizes in the upwelling region off Mauritania [27] revealed that ∼80% of the aggregates were larger than 0.5 mm, and 30–60% were larger than 1 mm in diameter. For this size distribution, the calculations clearly show that diffusion limited rates can be expected at concentrations below ∼20 µmol L^−1^. Rate measurements at low O_2_ are therefore challenged by the difficulty to reproduce insitu conditions with respect to shear stress and aggregate size, raising the question which incubation approach is best to meet these requirements. A thorough discussion of the optimal treatment is beyond the scope of this study and furthermore depends on the objectives of the incubation experiment. For example, determining half saturation constants for O_2_ respiration on a cellular level would require strong disaggregation and thus a high stirring speed, whereas assessing in situ consumption rates requires that shear stress is lowered to a minimum and in situ aggregate size distribution is maintained.

### Potential Detection Limits of Rate Measurements

The O_2_ consumption rates measured in all incubations were between 30 and 426 nmol L^−1^ h^−1^, which is comparable with previous rates measured in coastal shallow waters [11]. However, O_2_ consumption in the open Ocean, especially below the euphotic zone, is expected to be much lower, at approximately 1 nmol L^−1^ h^−1^ [9]. Assessing the potential of each method for measuring low rates, a rate detection limit for a 24 hour incubation was calculated from Eq 3. This rate detection limit is a potential limit as it is based only on sensor precision and measuring frequency, but neglects limitations from sensor drift and O_2_ contamination (see below). The potential rate detection limits ranged from 4.7 nmol L^−1^ h^−1^ (Exetainer, at high O_2_) to as low as 7 pmol L^−1^ h^−1^ (trace optode, at low O_2_), and most detection limits were 1–2 orders of magnitude below the target rate of ∼1 nmol L^−1^ h^−1^ (Table 1). All approaches show highest precision and lowest detection limits at low O_2_ concentrations. Especially both optodes show an almost linear decrease of the detection limit with decreasing O_2_ concentrations which is well explained by the non-linear response of the fluorescent signal to changing O_2_ concentrations [18]. Extrapolation of the detection limit to concentrations of 60 µmol L^−1^ (trace optode) and 200 µmol L^−1^ (full range optode) results in a detection limit of 0.5 nmol L^−1^ h^−1^ for both cases. Because the concentration dependent increase of detection limit is stronger for the trace optode compared to the full range optode the latter should be used for rate measurements at concentrations above 30 µmol L^−1^.

Given that the incubation time of 24 hours cannot be extended and that the precision, in case of MIMS and optodes, cannot be improved by the user, only the increase of measuring frequency can further decrease the detection limit. More Exetainers can be filled to increase the number of samples and the measuring frequency of the optodes can be increased to up to 1 s^−1^. The measuring frequency of the STOX sensor, however, is limited by its inherent response time [13], which is specific to the individual sensor. An increase of measuring frequency by shorter switching intervals of the front guard decreases the precision because of the exponential signal response after each switch. Nevertheless, technical improvements of the STOX could shorten the response time and thus the switching intervals.

### Specific Limitations of Rate Measurements

The rate detection limits discussed above are derived from statistical measures of short incubations with rather high consumption rates and should be treated as potential detection limits. There are other effects that may interfere with the measurements and increase the detection limit. Long term drift is a common problem of many sensors. The STOX sensor alternates each measurement with a zero calibration to reduce the effect of sensor drift. However, a remaining drift of up to 0.14% of the O_2_ concentration per hour was recently reported (Tiano et al. in review), which would result in a drift of 1.4 nmol L^−1^ h^−1^ at O_2_ concentrations of 1 µmol L^−1^. The detection limit of the STOX sensor may be therefore more than one order of magnitude higher due to sensor drift compared to the detection limit due to sensor precision and measuring frequency (0.08 nmol L^−1^ h^−1^ at 1 µmol L^−1^, Table 1). The optodes do not have a regular zero calibration, but rely on the high stability of their signal and calibration curve. A small drift in optode measurements is caused by photobleaching, i.e. the photochemical destruction of the fluorescent dye, which depends on the number of oxygen readings. According to the sensor specifications given by the manufacturer (www.presens.de) the drift was less than 0.002 and ∼0.05 nmol L^−1^ h^−1^, for the trace and full range optode, respectively, which is in the range of the calculated potential rate detection limits. However, photobleaching causes a decay in fluorescent intensity and affects fluorescent lifetime measurements to result in a positive drift, i.e. increasing O_2_ readings over time. The measured O_2_ consumption rates are therefore conservative estimates. For the MIMS measurement, a linear drift was considered using calibrations before and after the measurement. The good agreement between STOX and MIMS measurement in experiment 2 (Fig. 5) shows that a linear drift correction of the MIMS measurement (drift 0.8% h^−1^) was suitable.

Oxygen leakage into the incubation vial is another potential limitation for rate measurements, especially at low O_2_. In experiment 1+2, the diffusive O_2_ transport across the two ports of the incubation bottle can be neglected. Assuming O_2_ diffusivity of 1×10^−9^ m^2^ s^−1^ and a maximum O_2_ difference within the ports of 300 µmol L^−1^ a steady state flux would increase the O_2_ concentrations inside the incubation bottle by ∼0.08 nmol L^−1^ h^−1^, which is a very conservative estimate as the time to reach a steady state flux is not considered in this calculation. Oxygen contamination in anoxic incubations using Exetainers was reported recently by De Brabandere et al. [28] who measured O_2_ contaminations of 300–400 nmol L^−1^ introduced by butyl rubber septa of the caps of the Exetainers. In experiment 3, the residual concentrations of ^16-16^O_2_ were significantly higher (2 µmol L^−1^). The reason was most likely the high initial O_2_ concentration of the sea water used in this study, which apparently could not be completely removed by degassing with He for 15 minutes (see method section). Application of the Exetainer approach using low oxygen water from the Namibian OMZ (Kalvelage et al. in prep.) revealed residual O_2_ concentrations of less than 500 nmol L^−1^, measured with STOX sensor after degassing, filling and capping of the Exetainers. For future studies we recommend to use deoxygenated butyl rubber septa as described by De Brabandere et al. [28] to minimize O_2_ contamination.

In principal, the combined effect of sensor drift and O_2_ leakage rates can be estimated by a dead control incubation. In experiment 1, the incubated water was poisoned after 30 hours by adding 20 ml 60% ZnCl_2_. Interestingly, the STOX sensor and both optodes were still measuring O_2_ consumption rates between 5 and 30 nmol L^−1^ h^−1^ (Table 1). At O_2_ concentrations of 25 µmol L^−1^, a drift of the STOX sensor of 0.14% could explain the O_2_ decrease of 30 nmol L^−1^ h^−1^. However, the rates measured by the optodes cannot be explained by sensor drift, which would have resulted in increasing O_2_ rates of less than 0.1 nmol L^−1^ h^−1^. The remaining O_2_ consumption rather suggests that not all biological activity was terminated or that the O_2_ consumption was abiotic, eventually stimulated by the addition of ZnCl_2_. Instead of poisoning the sample after the incubation experiment we recommend running a control incubation using autoclaved, sterile and acidified water to quantify sensor drift and O_2_ leakage.

### Assets and Drawbacks of the Different Methods

The STOX sensor and the optodes provide continuous on-line measurements which allow detailed time series analysis as well as immediate correction and experimental adjustment. Furthermore, STOX sensor and optodes have the potential to detect very low rates (Table 2), because of their high precision (STOX) and high measuring frequency (optodes). In comparison, the rate detection limit is higher for the MIMS measurement, because the precision is less and the amount of individual samples is limited either by the amount of subsamples that can be pumped in the direct MIMS approach or the number of time points in the Exetainer approach. However, the methods with high precision and low rate detection limit (STOX, both optodes) rely on highly controlled measuring environments (optodes: stable temperature), on fragile and pricy sensors (STOX) and on extensive and often expensive periphery equipment, which requires considerable lab space on board or close to the sampling site. This limits the number of parallel incubations and replicates necessary to cover a large spatial heterogeneity in the field.

**Table 2:** Method comparison with respect to precision, detection limit and applicability.

Exp.	Method	Precision	Meas. Freq.	Pot. Det. Lim.	Fragility	Handling	Price
1	STOX	+++	++	+++	+	++	+
1	Optode (trace)	+++	+++	+++	+++	++	+++
1	Optode (full range)	++	+++	+++	+++	++	+++
2	MIMS (direct)	++	++	++	+	+	+++
3	MIMS (Exet.)	++	+	+	+++	+++	+++

The rating scheme is: regular (+), strong (++), and very strong (+++). Besides the precision, measuring frequency and potential rate detection limit, the sensor fragility, the handling in the field and the price of the consumables (sensor or label) are compared.

In contrast, the Exetainer incubations allow for a high number of parallel incubations, and to some extend the incubation in individual Exetainers already considers the heterogeneity of the water sample. The samples are fixed and collected during the field trip and can be processed later in the lab. Moreover, the Exetainer approach is easily combined with e.g. ^13^C and ^15^N labelling experiments [22] which allows investigating the C-cycle and N-cycle under dynamic oxygen conditions. This advantage holds especially for the direct MIMS measurement where oxygen consumption can be monitored with high temporal resolution in parallel to the production of gaseous N-species [29]. In general, there is great potential in combining any of the described O_2_ sensing methods with ^15^N labelling experiments to investigate the kinetics of nitrogen transformation processes (T. Dalsgaard et al. in prep.).

## Conclusions

In summary, all methods were suitable to detect O_2_ consumption rates in the range of a few nmol L^−1^ h^−1^ and at low O_2_ concentrations, representing rates and conditions as expected in the open Ocean and OMZs. Specific limitations of the rate measurements such as sensor drift (STOX) and oxygen contamination (Exetainer) need to be minimized to fully exploit the low detection limits derived from the sensor specific precision and measuring frequency. Potential detection limits for a 24 hour incubation were a few nmol L^−1^ h^−1^ or less, but differed among the methods. For continuous measurements and extremely low rates the STOX sensor and optodes are recommended, whereas the Exetainer/MIMS approach with ^18-18^O_2_ addition is recommended for many parallel incubations and simple handling in the field. A specific challenge for incubations at low O_2_ concentrations is the presence of aggregates in which diffusion limited rates can be expected at concentrations below ∼20 µmol L^−1^. To consider diffusion limited O_2_ consumption, a more refined sample treatment is needed that better reproduces in situ conditions with respect to aggregate size and distribution.

## Supporting Information

Appendix S1 **Mathematical derivation of** **equation (3** **) for calculating the Standard Error from measuring frequencies and incubation times.**
(DOC)
