# Supplementary material for: Aquatic Respiration Rate Measurements at Low Oxygen Concentrations
Source: PLoS One. 2014 Feb 19;9(2):e89369. doi: 10.1371/journal.pone.0089369 (PMC3929708; doi:10.1371/journal.pone.0089369)
Supplement: Appendix S1 — Mathematical derivation of equation (3 ) for calculating the Standard Error from measuring frequencies and incubation times. (DOC) [file pone.0089369.s001.doc]

**S1 Appendix**

The standard error of the slope of the linear regression is given by:

$SE=\frac{RMS_{RES}}{\sqrt{\sum_{i=1}^{n} \left(t_{i}-\bar{t}\right)^{2}}}$ (A1)

where n is the number of measurements. The series in the denominator of (A1) can be rearranged to

$\sum_{i=1}^{n} \left(t_{i}-\bar{t}\right)^{2}=\left(\sum_{i=1}^{n} t_{i}^{2}\right)-\frac{1}{n}\left(\sum_{i=1}^{n} t_{i}\right)^{2}$ (A2)

The two series on the right side of (A2) can be simplified when introducing the period P between the measurements t_i_ and t_i+1_:

$\left(\sum_{i=1}^{n} t_{i}^{2}\right)=P^{2}\frac{n(n+1)(2n+1)}{6}$ (A3a)

$\frac{1}{n}\left(\sum_{i=1}^{n} t_{i}\right)^{2}=\frac{1}{n}P^{2}\left(\frac{n(n+1)}{2}\right)^{2}$ (A3b)

Insertion of (A3a) and (A3b) into (A2) and considering that the time for the entire incubation is expressed as $T=P n$, results in

$\sum_{i=1}^{n} \left(t_{i}-\bar{t}\right)^{2}=\frac{T^{2}}{12}\left(n-\frac{1}{n}\right)$ (A4)

For large n (A4) converges to:

$\sum_{i=1}^{n} \left(t_{i}-\bar{t}\right)^{2}=\frac{T^{2} n}{12}$ (A5)

Insertion of (A5) into (A1) and introducing the measuring frequency $F=n/T$results in

$SE\approx\frac{3.46 RMS_{RES}}{T\sqrt{TF}}=3.46 RMS_{RES} T^{-1.5}F^{-0.5}$ (A6)
